# Development of droplet digital Polymerase Chain Reaction assays for the detection of long-finned (*Anguilla dieffenbachii*) and short-finned (*Anguilla australis*) eels in environmental samples

**DOI:** 10.7717/peerj.12157

**Published:** 2021-09-27

**Authors:** Georgia Thomson-Laing, Russleigh Parai, Laura T. Kelly, Xavier Pochon, Rewi Newnham, Marcus J. Vandergoes, Jamie D. Howarth, Susanna A. Wood

**Affiliations:** 1 Cawthron Institute, Nelson, New Zealand; 2 Victoria University of Wellington, Wellington, New Zealand; 3 Institute of Marine Science, University of Auckland, Warkworth, New Zealand; 4 GNS Science, Lower Hutt, New Zealand

**Keywords:** Environmental DNA, eDNA, Metabarcoding, High-throughput sequencing, *Anguilla*, droplet digital PCR

## Abstract

Freshwater eels are ecologically, and culturally important worldwide. The New Zealand long-finned eel (*Anguilla dieffenbachii*) and short-finned eel (*Anguilla australis*) are apex predators, playing an important role in ecosystem functioning of rivers and lakes. Recently, there has been a national decline in their populations due to habitat destruction and commercial harvest. The emergence of targeted environmental DNA detection methodologies provides an opportunity to enhance information about their past and present distributions. In this study we successfully developed species-specific droplet digital Polymerase Chain Reaction (ddPCR) assays to detect *A. dieffenbachii* and *A. australis* DNA in water and sediment samples. Assays utilized primers and probes designed for regions of the mitochondrial cytochrome b and 16S ribosomal RNA genes in *A. dieffenbachii* and *A. australis*, respectively. River water samples (*n* = 27) were analyzed using metabarcoding of fish taxa and were compared with the ddPCR assays. The presence of *A. dieffenbachii* and *A. australis* DNA was detected in a greater number of water samples using ddPCR in comparison to metabarcoding. There was a strong and positive correlation between gene copies (ddPCR analyses) and relative eel sequence reads (metabarcoding analyses) when compared to eel biomass. These ddPCR assays provide a new method for assessing spatial distributions of *A. dieffenbachii* and *A. australis* in a range of environments and sample types.

## Introduction

Documenting changes in biodiversity is becoming increasingly important due to the exponential rise in species losses at local, regional, and global scales (*e.g.*, Butchart et al., 2010; Dirzo & Raven, 2003; He et al., 2017). In fresh-water ecosystems, traditional surveillance for fish uses nets, carrion-baited traps, visual surveys, or electrofishing to obtain an overview of the existing community (Joy, David & Lake, 2013). These techniques are costly, labor and time intensive, and the detection of rare species requires a high sampling effort. Traditional methods can be environmentally invasive, often resulting in bycatch and require direct handling of target organisms (Portt et al., 2006; Reynolds & Holliman, 2004).

The application of molecular techniques to detect environmental DNA (eDNA) in a range of sample types forgoes many limitations of traditional surveying (reviewed by Senapati et al., 2019). The concept of species detection based on eDNA relies on the assumption that all organisms release their DNA (*i.e.,* through decomposition, skin cell shedding, waste production) to a collective pool of DNA that exists in the physical environment. Assays analyzing eDNA can be either designed for a specific target, such as a single species (or taxa) or non-targeted to assess an entire biological community.

Target-specific eDNA detection techniques have been applied to a range of aquatic vertebrates and invertebrates, including abundant, rare, invasive, and endangered taxa. Detecting eDNA of specific species can be more sensitive than traditional practices, especially when the organisms are at low densities, *i.e.,* rare species in large water bodies (*e.g.*, Jerde et al., 2011; Sigsgaard et al., 2015; Takahara, Minamoto & Doi, 2013; Wilcox et al., 2016). Quantitative real-time PCR (qPCR) assays have been shown to be a sensitive and quantitative approach to detect aquatic organisms, *i.e.,* fish (*e.g.*, Atkinson et al., 2018; Laramie, Pilliod & Goldberg, 2015; Olsen et al., 2015; Piggott, 2017; Sigsgaard et al., 2015; Takahara, Minamoto & Doi, 2013; Turner et al., 2014; Wilcox et al., 2013), invertebrates (*e.g.*, Goldberg et al., 2013; Mauvisseau et al., 2018; Tréguier et al., 2014) and amphibians (*e.g.*, Pilliod et al., 2013; Secondi et al., 2016; Smart et al., 2015). Recently, the development of droplet digital PCR (ddPCR), which measures absolute DNA copy numbers, has further increased assay sensitivity, especially in the presence of PCR inhibitors (Doi et al., 2015a; Doi et al., 2015b; Mauvisseau et al., 2019b; Simmons et al., 2015). Some studies that use quantitative methods (*i.e.,* qPCR and ddPCR) have shown positive correlations of PCR copy numbers to the abundance and/or biomass of the target organism in a waterbody (Hinlo et al., 2017; Klobucar, Rodgers & Budy, 2017; Mizumoto et al., 2018).

Metabarcoding is increasingly being used to characterize the species diversity of aquatic communities (Blackman et al., 2017; Hänfling et al., 2016; Klymus, Marshall & Stepien, 2017; Shaw et al., 2016; Valentini et al., 2016). In contrast to target-specific approaches such as ddPCR, metabarcoding enables the simultaneous identification of many species and thus the community composition of groups of organisms, *e.g.* eukaryotes. However, many studies recognize the various challenges associated with the amplification of multi-template sequences. For example, primers are often not conserved across the entire community of interest and therefore not universal, primers can also be biased to certain organisms or species and reference databases remain inaccurate and often incomplete leading to incorrect or incomplete taxonomic assignment of sequences (Clarke et al., 2014; Deagle et al., 2014; Dowle et al., 2016; Wangensteen et al., 2018). Recently, universal primer sets and PCR assays for metabarcoding fishes have been developed (MiFish-U/E and Teleo-F/R; Miya et al., 2015; Valentini et al., 2016). Each primer set targets a section within the 12S rRNA gene; Mifish-U-F and Mifish-U-R (Miya et al., 2015) targets a region of approximately 220 base pairs (bp), while Teleo-F and Teleo-R (Valentini et al., 2016) targets a different region of approximately 100 bp. Both primer sets have sufficient coverage to detect a wide range of fishes in various habitats; marine (Miya et al., 2015), lakes (Fujii et al., 2019) and rivers (Doi et al., 2019; Valentini et al., 2016). However, *in silico* and *in vitro* trials of the two primer pairs have previously highlighted differences in their ability to distinguish New Zealand freshwater fish species (Banks, Kelly & Clapcott, 2020). Only a limited number of studies have compared detection rates between qPCR or ddPCR and metabarcoding approaches, with most recommending application of a targeted approach when sensitive detection of a specific species is paramount (*e.g.*, Wood et al., 2019). A further significant advantage of qPCR or ddPCR assays over metabarcoding, is that the results are instantaneous post PCR, whereas metabarcoding samples require high-throughput sequencing post PCR and bioinformatic processing. The cost of high-throughput sequencing machines is often prohibitive meaning they are sent to specialized laboratories, adding to the length of time for results to be returned.

In freshwater environments, the analysis of eDNA in water samples is commonly used in preference to sediment samples because there is generally a greater probability that eDNA from fish and other vertebrates will be detected in these samples (*e.g.*, Baldigo et al., 2017; Buxton, Groombridge & Griffiths, 2018; Shaw et al., 2016). However, some studies indicate that eDNA is found at higher, albeit more variable, concentrations and persists for longer in aquatic sediments in comparison to water (Eichmiller, Bajer & Sorensen, 2014; Sakata et al., 2020; Turner, Uy & Everhart, 2015). Sediments can also act as a sink for DNA, expanding the timescale at which eDNA can be assessed (Sakata et al., 2020). A number of studies have highlighted the potential for reconstructing historical trends in catchment use, species colonization history and aquatic community composition by eDNA analysis of terrestrial plants and animals (Giguet-Covex et al., 2014; Parducci et al., 2019; Pedersen et al., 2016), and freshwater fishes (Nelson-Chorney et al., 2019; Olajos et al., 2018) in lake sediment cores.

Freshwater eels (*Anguilla* sp.) have large economic, cultural and ecological importance worldwide, but global stocks are declining (Arai, 2014; Castonguay & Durif, 2015). Three freshwater eel species are found in New Zealand. The endemic long-finned eel (*Anguilla dieffenbachii*) and the native short-finned eel (*Anguilla australis*) are widespread throughout rivers and lakes. The Australian speckled long-finned eel (*A. reinhardtii*) inhabits a small western region of the North Island (Jellyman et al., 1996). In freshwater food webs, eels are the apex predator, and they play an important role in ecosystem functioning (*e.g.*,  Kelly & Jellyman, 2007). Additionally, *A. dieffenbachii* and *A. australis* support important traditional and commercial fisheries in New Zealand (Jellyman, 2007). These eels (or tuna, as the are known by Mãori, the indigenous people of New Zealand) are harvested by Mãori and represent an important part of their cultural history, often featuring in their mythology (Doole, 2005; Jellyman, 2007). Although still common, there has been a national decline in eel populations, especially *A. dieffenbachii* due to habitat destruction (*i.e.,* installation of dams, weirs and wetland loss) and commercial take (Beentjes, Jellyman & Kim, 2006; Boubee et al., 2003; Doole, 2005; Hoyle & Jellyman, 2002; Jellyman, 2007; Jellyman et al., 2000). Climate change has also been implicated as a future risk to eel recruitment in New Zealand (August & Hicks, 2008). Eel populations in New Zealand are dependent on a successful reproductive life cycle, characterized by long-distance migrations between fresh- and seawater environments where feeding and growth, and spawning occur, respectively. The life histories of both New Zealand eels remain enigmatic, with the exact location of spawning grounds in the Pacific Ocean not yet known (Jellyman & Tsukamoto, 2002).

Historically, eel population size and distribution have been determined *via* a range of different capture methods, *e.g.*, baited or unbaited traps, fyke netting, beam trawls or electrofishing (*e.g.*, Beentjes, Jellyman & Kim, 2006; Jellyman, 1996; Jellyman & Chisnall, 1999; Jellyman & Graynoth, 2005). Species-specific probe-based qPCR assays have been successfully developed for a range of freshwater eels globally, namely the European eel, *Anguilla anguilla* (Weldon et al., 2020), giant mottled eel *Anguilla maramorata* (Itakura et al., 2020), and Japanese eel *Anguilla japonica* (Watanabe et al., 2005). These *Anguilla* sp. specific assays have been used successfully in rivers (Itakura et al., 2020; Itakura et al., 2019), lakes (Weldon et al., 2020), and experimental tanks*.* Comparisons between quantitative eDNA methods and fishing surveys have highlighted the reliability and sensitivity of these eDNA methods and there are weak correlations between eDNA concentration with the abundance and biomass of eels (Itakura et al., 2020; Itakura et al., 2019; Weldon et al., 2020). This study aimed to develop species-specific molecular assays that could be used for the detection of *A. dieffenbachii* and *A. australis* in environmental water and sediment samples.

## Materials & Methods

### Primer/probe design and *in silico* specificity

Species-specific assays were designed *in silico* for *A. australis* and *A. dieffenbachii*. The *A. australis* assay targeted the mitochondrial 16S ribosomal RNA (16S rRNA) gene and the *A. dieffenbachii* assay targeted the mitochondrial *cytochrome b* (*cytb*) gene. Nucleotide sequences of *A. australis* and *A. dieffenbachii* (16S *rRNA* and *cytochrome b* genes) were sourced from the National Centre for Biotechnology Information nucleotide database (NCBI; https://www.ncbi.nlm.nih.gov/; Tables S1 and S2). Primers and probes were designed using Primer3 (Untergasser et al., 2012) from a consensus alignment of multiple sequences (Tables S1 and S2) to reduce potential intraspecific variability. In addition, target amplicons were aligned *in silico* with a wider range of *Anguilla* spp. (Tables S1 and S2) to determine percent similarity of sequences and to check for interspecific cross-reactivity. Target amplicons were also blasted against a wider database (Blastn; NCBI) to further check that no cross-reactivity would occur with other fish species. Primetime TaqMan probes and molecular beacon probes (IDT) were used for *A. australis* and *A. dieffenbachii,* respectively. Both probes are oligonucleotides that hybridize to an internal region of the PCR product and release fluorescence during PCR, but unlike TaqMan probes that release fluorescence during replication through cleavage, molecular beacons use changes in structure to cause fluorescence and therefore remain intact during PCR and must rebind to the target in every cycle, which makes the probes more sensitive to single-base mismatches. To maximize assay specificity, primers and probes were designed in regions of the genes exhibiting the most interspecific variability among all eel species found in New Zealand (*A. australis*, *A. dieffenbachii* and *A. reinhardhtii*; Tables S1 and S2). The design specifically focused on identifying nucleotide mismatches among species at the 3′end of the primer.

### Sample collection

#### Tissue samples

The specificity of both assays was tested on DNA extracted from tissue from *A. dieffenbachii* and *A. australis*, as well as a range of freshwater fish species commonly found in New Zealand. Tissue samples from morphologically identified *A. australis* and *A. dieffenbachii* specimens were provided from other projects. The samples were collected from Whakaki Lagoon (39°02′45″S, 177°32′50″E) or Te Waihora/Lake Ellesmere (43°47′21″S, 172°27′19″E), and the Maitai River (41°16′49″S, 173°19′47″E), respectively. All tissue samples were collected under the specifications of Special Permit 651 from the New Zealand government agency Ministry for Primary Industries.

The specificity of the assays was tested using DNA from other New Zealand freshwater/brackish fish species which were collected as described in Brjkic & Lear (2017). Species tested included giant kokopu (*Galaxias argenteus*), black mudfish (*Neochanna diversus*), estuarine triplefin (*Forsterygion nigripenne*), Cran’s bully (*Gobiomorphus basalis*), upland bully (*Gobiomorphus breviceps*), giant bully (*Gobiomorphus gobioides*), bluegill bully (*Gobiomorphus hubbsi*), redfin bully (*Gobiomorphus huttoni*) and shortjaw kokupu (*Galaxias postvectis*).

#### Environmental samples

Water samples were collected from 11 rivers across New Zealand (Table S3). Water samples (*n* = 1 to 5 per site) were collected to compare metabarcoding and ddPCR methods across a range of rivers and sites. For a subset of these sites (W9–W13), water samples were collected in triplicate to compare metabarcoding and ddPCR with eel biomass.

Single point water samples (0.25–10 L) were collected mid-river using a Smith-Root eDNA backpack sampler (ANDe™ system; Thomas et al., 2018) or Geotech pump system and filtered using Polyethersulfone (PES) membrane filters (1.2 µm or 5 µm; Table S3). One liter of sterile water was filtered in the field as a control for onsite contamination (sample W11). Filters were transferred to sterile tubes and stored at −20 °C (<3 weeks) before DNA extraction and subsequent ddPCR and high-throughput sequencing (HTS).

Within one day of water sample collection at sites W9–W13, fish biomass assessments were also carried out. In a 150 m stretch of river, fish were caught by electrofishing as per Joy, David & Lake (2013), taxonomically identified, counted and length measured. Fish weight in grams (W) was calculated by W = aL^b^, where L is fish length (cm), *a* is the intercept and *b* is the slop value estimated from a linear regression of log-transformed length-weight data (Jellyman et al., 2013). Total fish biomass was calculated at each site and used to determine both total and relative biomass of *A. dieffenbachii* and *A. australis*. Eels that could not be identified to species level were classified as unidentified *Anguilla*. Two of these sites, W9 and W10, had no eel biomass recorded and were therefore considered negative field control sites.

Surface sediment samples were collected from three sites in the upper South Island: Lake Rotoiti, Maitai River and Tasman Valley Stream (Table S4). These locations were chosen due to previous knowledge and observations of high concentrations of *A. dieffenbachii* and/or *A. australis*. At each location, a combination of surface sediment (<2 cm depth) and biofilm (removed from rock surfaces) samples (*n* = 5 to 7) were collected using a sterile spatula and stored in sterile tubes at −20 °C (<1 week) before DNA extraction and subsequent ddPCR analysis (Table S4)

### DNA extraction

All molecular analyses (DNA extractions and PCRs) were conducted in sterile laboratories, with separate and sequential workflow to reduce cross-contamination. Benchtop UV sterilisation (>15 min) was undertaken before DNA extractions and PCR set-up. PCR set-up was done in laminar flow cabinets with HEPA filtration.

DNA was extracted from tissue samples using the DNeasy^®^ Blood and Tissue Kit (QIAGEN, USA) following the manufacturer’s instructions for tissue samples. DNA was extracted from the PES filters using the Zymo Blood and Tissue Kit according to the manufacturer’s directions. As preliminary experiments indicated that inhibition was present in most samples, all DNA samples were diluted 1 in 10 prior to downstream analysis. DNA was extracted from sediment samples using the DNeasy PowerSoil^®^ DNA Isolation Kit (QIAGEN, USA). A subsample of surface sediment was weighed directly into the first tube of the kit and the extraction performed following the manufacturer’s protocol. A blank extraction without a sample was undertaken using only extraction kit buffers for all sample types.

### Droplet digital PCR

Absolute concentrations of the mitochondrial 16S rRNA and *cytb* genes for *A. australis* and *A. dieffenbachii* respectively, were measured in tissue and environmental samples using a BioRad QX200 ddPCR system. Each ddPCR reaction had a total volume of 22 µL and included primers (forward and reverse; 454 nM), probe (454 nM), 1 × BioRad ddPCR Supermix for probes (no dUTP), 1–3 µL DNA, and sterile water. The ddPCR reaction mixture (20 µL) was combined with 70 µL of BioRad droplet oil for probes and partitioned into nanodroplets by the BioRad QX200 droplet generator. The nanodroplet emulsion (40 µL) was transferred to and amplified in a PCR plate using the following cycling protocol; 95 °C for 10 min for initial denaturation, 45 cycles of 94 °C (30 s) and 59 °C (1 min; selected after testing different annealing temperatures), and a final step of 98 °C, 10 min for enzyme deactivation. The QX200 droplet reader (BioRad) was then used to analyze the plate. For each ddPCR assay, at least one negative methodological control (RNA/DNA-free water Life Technologies), one negative biological control (1 ng µL^−1^ tissue DNA extracted from non-target eel species) and one positive control (1 ng µL^−1^ tissue DNA extracted from target eel species) were included.

Fluorescence amplitude thresholds for positive droplets were determined separately for each assay (10,000 and 2,000 amplitude for *A. dieffenbachii* and *A. australis* assays, respectively) based on the amplitude of negative droplets across both methodological and biological negative controls. For quality control, no positive droplets were allowed in either negative control for assay results to be accepted. When a single positive droplet occurred in a well, the sample was run twice more to confirm if the sample was positive (droplet in two of the triplicates) or negative (droplet only in one of the triplicates).

### Estimation of assay limit of detection and quantification

Synthetic sections of target DNA (gblocks; manufacturer requirement to be >125 bp, Integrated DNA Technologies) were designed to match the *A. australis* 16S rRNA gene amplicon sequence (126 bp; including 21 additional bases on each end of the amplicon; Table S5) and the *A. dieffenbachii cytb* gene amplicon sequence (138 bp; including 6 additional bases on each end of the amplicon; Table S5). The highest concentrations of gblocks and target tissue DNA were quantified (ng µL^−1^) using a Qubit (ThermoFisher Scientific, USA).

The ddPCR assay limits of detection (LOD) and quantification (LOQ) for tissue DNA were estimated using a ten-fold dilution series (in duplicate) of target tissue DNA ranging from 1 ng µL^−1^ to 0.1 fg µL^−1^. The LOD was defined as the last standard dilution at which the targeted DNA was detected and quantified in at least two out of three replicates. The LOQ was defined as the last standard dilution in which the targeted DNA was detected and quantified in all replicates.

Assay accuracy was tested by calculating % yield from gblocks (formula below) using ten-fold dilution series ranging from 6000–0.0006 copies uL^−1^ and 10,000–0.001 copies uL^−1^ for *A. australis* and *A. dieffenbachia*, respectively. Copies per well (of gblocks) was calculated from a known concentration (ng) using the molecular weights (provided by manufacturer) of the target amplicons for *A. australis* (77,716 g mol^−1^) and *A. dieffenbachii* (85,134 g mol^−1^).

\begin{eqnarray*}\text{%}~yield~from~gblocks= \frac{number~of~copies~ \left( per~well \right) ~measured}{number~of~copies~(per~well)~expected} \times 100. \end{eqnarray*}

### Sanger sequencing

Amplicon sequence confirmation was carried out on DNA from *A. australis* and *A. dieffenbachii* tissue samples, as well as environmental samples to confirm assay specificity (see Table 1). For sequencing preparation, ddPCR product was pooled and cleaned based on the manufacturer suggested protocol (Droplet Digital Application Guide; “Amplicon Recovery from Droplets”; http://www.bio-rad.com/webroot/web/pdf/lsr/literature/Bulletin_6407.pdf; BioRad). Briefly, ddPCR reactions were carried out for each assay separately as previously described. Samples were assayed using 2 to 10 times dilutions depending on the amplicon concentration. Droplets for one replicate were read as per the normal protocol to confirm the successful amplification of ddPCR product. Prior to droplet analysis, the full well volume (40 µL) of all other replicate samples were transferred to a new tube.

**Table 1:** Droplet digital PCR and high-throughput sequencing of *Anguilla* DNA extracted from river water. Droplet digital PCR (ddPCR) amplification with *Anguilla australis* and *Anguilla dieffenbachii* specific assays and high-throughput sequencing (HTS) of DNA extracted from river water samples.

Site ID	Sample size	Species	HTS relative abundance (% of total fish community)		ddPCR (copies µL^−1^)	
			**Average (±se)**	**Positive detection/** **samples analyzed**	**Average (±se)**	**Positive detection/** **samples analyzed**
W1	*n* = 1	*A. australis*	44%	1/1	0.75	1/1
		*A. dieffenbachii*	55%	1/1	0.24	1/1
W2	*n* = 1	*A. australis*	17%	1/1	0.085	1/1
		*A. dieffenbachii*	–	0/1^b^	0.2	1/1^b^
W3	*n* = 1	*A. australis*	62%	1/1	0.51	1/1
		*A. dieffenbachii*	–	0/1	–	0/1
W4	*n* = 1	*A. australis*	60%	1/1	1.03	1/1
		*A. dieffenbachii*	21%	1/1	0.06	1/1
W5	*n* = 1	*A. australis* ^a^	14%	1/1	2.6	1/1
		*A. dieffenbachii* ^a^	9%	1/1	4.33	1/1
W6	*n* = 1	*A. australis* ^a^	7%	1/1	5.21	1/1
		*A. dieffenbachii*	2%	1/1	0.34	1/1
W7	*n* = 1	*A. australis*	1%	1/1	0.02	1/1
		*A. dieffenbachii*	29%	1/1	0.38	1/1
W8	*n* = 3	*A. australis*	–	0/3^b^	0.12	1/3^b^
		*A. dieffenbachii*	75 ± 11.3%	2/3^b^	0.51 ± 0.21	3/3^b^
W9	*n* = 3	*A. australis*	–	0/3	–	0/3
		*A. dieffenbachii*	12.6%	1/2^b^	0.56 ± 0.36	3/3^b^
W10	*n* = 3	*A. australis*	–	0/3	–	0/3
		*A. dieffenbachii*	–	0/3^b^	0.07	1/3^b^
W11	*n* = 5	*A. australis*	13.0 ± 2.64%	5/5	4.69 ± 0.47	5/5
		*A. dieffenbachii*	52.9 ± 2.00%	5/5	12.62 ± 0.93	5/5
W12	*n* = 3	*A. australis*	77.7 ± 3.91%	3/3	4.82 ± 0.44	3/3
		*A. dieffenbachii*	16.9 ± 2.75%	3/3	1.26 ± 0.37	3/3
W13	*n* = 3	*A. australis*	15.0%	1/3^b^	1.01 ± 0.16	3/3^b^
		*A. dieffenbachii*	10.6 ± 0.36%	2/3^b^	1.74 ± 0.31	3/3^b^
W14 (control)	*n* = 1	*A. australis*	–	0/1	–	0/1
		*A. dieffenbachii*	–	0/1	–	0/1

**Notes.**

a ddPCR products were sequenced and confirmed amplification of correct sequence.

b samples with different number of positive detections between HTS and ddPCR methods.

After all droplets floated to the top, the floating droplet phase was retained from the ddPCR product mix and the bottom oil phase discarded. An aliquot of TE buffer (20 µL for 1× ddPCR well, total 40 µL > 1 ddPCR well) was added to the droplet phase, followed by chloroform (70 µL for 1× ddPCR well, total 140 µL > 1 ddPCR well). The mix was vortexed (1 min) and centrifuged (15,500× g, 10 min). The ddPCR amplicon (upper aqueous layer) was retained, quantified (Qubit) and kept at −4 °C prior to sequencing. Bi-directional sequencing was undertaken using the BigDye Terminator v3.1 Cycle Sequencing Kit at the Genetic Analysis Services, University of Otago (Applied Biosystems, CA, USA).

### High-throughput sequencing and bioinformatics

For water samples (W1–W8, W14), regions of the mitochondrial 12S rRNA gene were amplified using two previously published primer sets with illumina tags: MiFish-UF and MiFish-UR (Miya et al., 2015) and Teleo-R and Teleo-F (Valentini et al., 2016). For samples W9–W13, only the MiFish primer set was used. Each PCR reaction consisted of 10 µL of 2 × MiFi Taq Mastermix (Bioline, London, UK), 1 µL of the relevant forward and reverse primer, 6 µL of DNAse free sterile water (Invitrogen, Carlsbad, CA, USA) and 2 µL of template DNA. Each PCR run included a positive control (DNA extracted from the tissue of *G. argenteus*) and a no template control. Cycling conditions consisted of an initial denaturation step at 95 °C for 2 min, followed by 40 cycles of denaturation at 95 °C for 30 s, annealing at 55 °C for 30 s and extension at 72 °C for 45 s, with a final extension at 72 °C for 5 min. Each PCR was conducted in triplicate to minimize the impact of PCR biases and the PCR product pooled for visualization on a 1% agarose gel. The pooled PCR product was purified and normalized using SequelPrep Normalization plates (Applied Biosystems, Foster City, CA, USA), resulting in a concentration of ∼1 ng mL^−1^. The cleaned samples were sent to Auckland Genomics Facility for paired-end sequencing on an Illumina Miseq™ platform (2 × 250 bp and 1 × 150 bp for MiFish and Teleo assays, respectively). The concentration and quality of the library was quantified using a bioanalyzer. The library was diluted to 4 nM, denatured and a 15% PhiX spike added. The library was further diluted to a final loading concentration of 7 pM. Raw sequence reads are deposited in the NCBI short read archive (SRP319777).

Primers were removed from the raw reads with the program Cutadapt (Martin, 2011) allowing one mismatch. Sequences without primer sequences were discarded. Remaining sequences were processed with DADA2 (Callahan et al., 2016) within the R framework (R Core Team, 2016). Sequences were filtered and trimmed to 150 bp for the MiFish primer set and 85 bp for the Teleo primer set, with a maximum expected error of two for forward reads and four for reverse reads. Error profiles for both forward and reverse reads were estimated with DADA2 using 10^8^ bases. Sequences were then dereplicated and sample inference undertaken for each sample. Forward and reverse reads were merged with a maximum of one mismatch and a minimum overlap of 50 bp for the MiFish sequences and 40 bp for the Teleo sequences. Sequences were size-selected (160–240 nucleotides for MiFish and 90–140 nucleotides for Teleo), and chimeras were removed using the removeBimeraDenovo command in DADA2. A reference database was constructed using 12S rRNA sequences of chordates downloaded from GenBank and supplemented with 12S rRNA sequences of New Zealand native fishes (Table S6; Banks, Kelly & Clapcott, 2020). Taxonomic assignment was undertaken using DADA2 and the assign Taxonomy command with bootstrapping increased to 90. This was undertaken due to the closely related nature of many of New Zealand’s freshwater species to reduce the risk of spurious species assignments. The number of reads for amplicon sequence variants (ASVs) present in the negative controls was subtracted from all samples. The resulting ASVs with the corresponding taxonomic assignment were filtered to exclude non-fish sequences. Samples with <100 reads were removed. Read abundance tables for *Anguilla* spp. were constructed from the data using the Phyloseq package (McMurdie & Holmes, 2013) in Rstudio (R Studio Team, 2015). Read numbers were converted into relative read abundance (% of total fish abundance) and when both primer sets were used, results were averaged from Teleo and MiFish primer sets.

### Data analysis

Data distributions were evaluated with exploratory histograms and boxplots to ensure assumptions of normality and homogeneity of variance (Levene’s test) were met. DNA concentrations (from eel tissue and gblocks), copy numbers of amplicons, relative eel reads from metabarcoding and eel biomass parameters were log-transformed prior to analysis to normalize the data. Simple linear regression was undertaken to determine standard curve correlations between dilution series of a known amount of DNA (from eel tissue or gblocks) and copy numbers of amplicons. For environmental data, simple linear regressions were used to determine relationships between log-transformed biomass parameters (relative and total), relative eel reads from metabarcoding and copy numbers of amplicons in a subset of water samples (W11–W13) with sufficient biological replication (*n* = 3 or *n* = 5). Data from negative sites W9 and W10 were predominantly zero values and these were excluded from linear regression analyses. Statistical analyses were conducted using R software (R Core Team, 2016; R Studio Team, 2015) with ggplot2 (Wickham, 2016) and heplots (Fox, Friendly & Monette, 2018).

## Results

### Primer/probe design and *in silico* specificity

**Figure 1:**
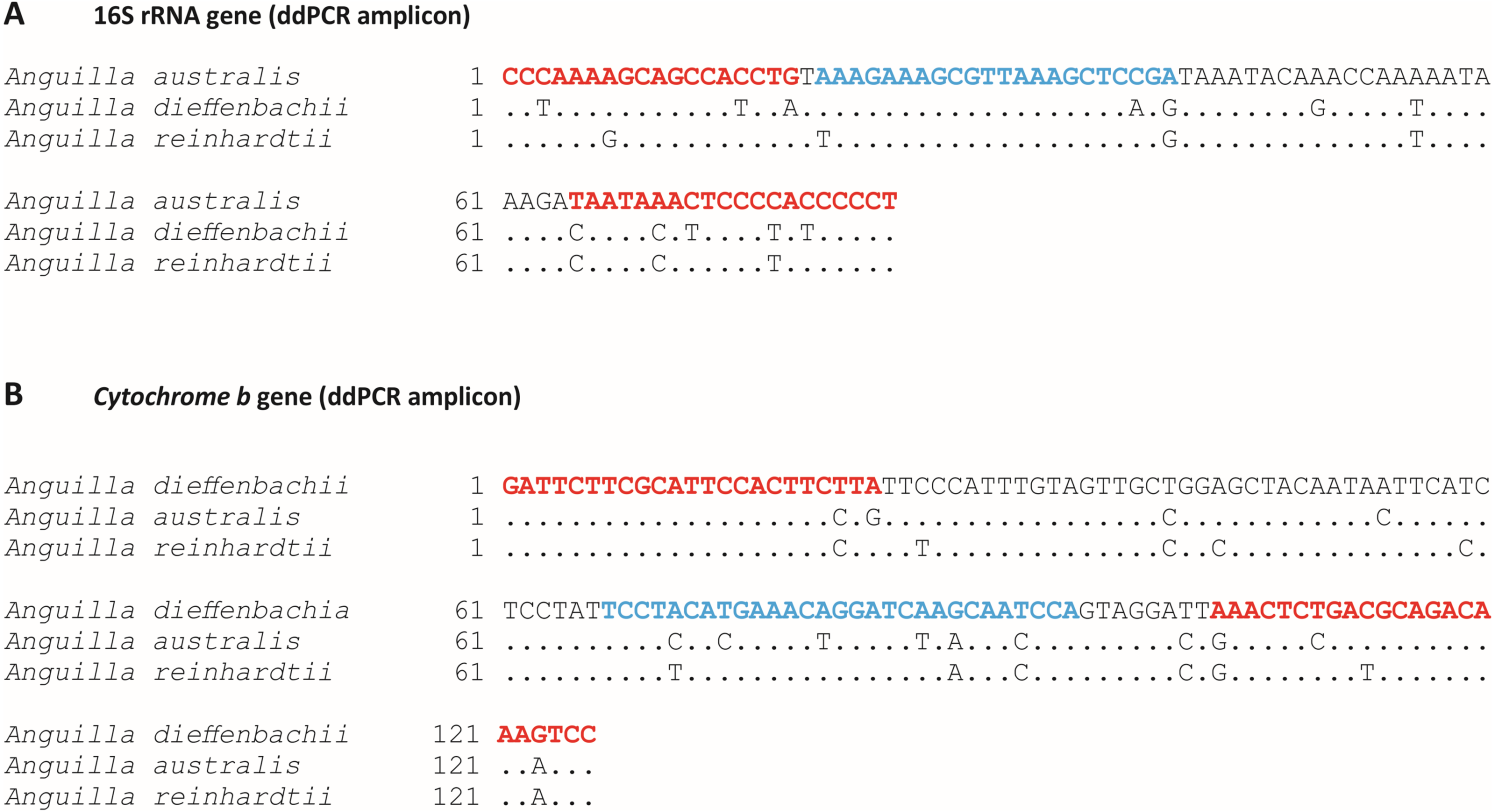
Target species-specific primer and probe sequences used for amplification of *Anguilla australis* or *Anguilla dieffenbachii* DNA by droplet digital PCR. Target species-specific sections of; (A) 16S ribosomal RNA gene, and (B) *Cytochrome b* mitochondrial gene for species-specific amplification by droplet digital PCR (ddPCR) for; (A) *Anguilla australis*, and (B) *Anguilla dieffenbachii*. Species-specific primer and probe positions are indicated by red and blue text, respectively. Interspecific sequence mismatches between New Zealand *Anguilla* spp. are shown.

The *A. australis* assay amplified a 126 bp region of the 16S rRNA mitochondrial gene using the forward primer (A.aust16S-F: 5′–CCC AAA AGC AGC CAC CTG –3′), reverse primer (A.aust16S-R: 5′–AGG GGG TGG GGA GTT TAT TA –3′) and primetime probe (A.aust16S-P: 5′–/56-FAM/AAA GAA AGC/ZEN/GTT AAA GCT CCG A/3IABkFQ/ –3′; Fig. 1A). The *A. dieffenbachii* assay amplified a 138 bp region of the *cytb* mitochondrial gene using the forward primer (A.dieffCytB-F: 5′–GAT TCT TCG CAT TCC ACT TCT TA –3′), reverse primer (A.dieffCytB-R: 5′–GGA CTT TGT CTG CGT CAG AGT TT –3′) and molecular beacon probe (A.dieffCytB-P: 5′–/56-FAM/TCC TAC ATG AAA CAG GAT CAA GCA ATC CA/3IABkFQ/ –3′; Fig. 1B). The sequence similarity of *A. dieffenbachii* and *A. australis* for the whole 16S rRNA and *cytb* genes, was 97% and 94% respectively. Sequence similarity of amplified products between the two species was 86% and 89% for 16S rRNA and *cytb*, respectively (Tables S1 and S2).

*In silico* specificity was validated by nucleotide mismatches between species-specific primer and probes and non-target *Anguilla* sp. Specifically, there were 10 base pair mismatches (across both primers and probe) between the *A. australis* assay and the *A. dieffenbachii* gene sequence (Fig. 1A) and similarly there were 11 base pair mismatches between the *A. dieffenbachii* assay and the *A. australis* gene sequence (Fig. 1B). In addition, *in silico* testing identified six and seven base pair mismatches between the *A. reinhardtii* gene sequence and the *A. australis* and *A. dieffenbachii* assays, respectively.

### Assay validations using ddPCR

#### Assay specificity

*Anguilla australis* and *A. dieffenbachii* ddPCR assays successfully amplified tissue DNA from morphologically identified *A. australis* and *A. dieffenbachii* specimens (Table 1). There was a distinctive division between positive and negative droplets in both ddPCR assays (Fig. S1). There was no cross-reactivity between assays for each eel species and tissue DNA from the non-target eel species at the maximum DNA concentrations tested (1 ng µL^−1^; Fig. S1). Sequencing confirmed the correct amplification of either the *A. australis* 16S rRNA gene or *A. dieffenbachii cytb* gene in all ddPCR products that were sent for sequencing. Neither eel ddPCR assay cross-reacted with any of the non-target freshwater fish species assessed.

#### Assay sensitivity and percentage yield

**Figure 2:**
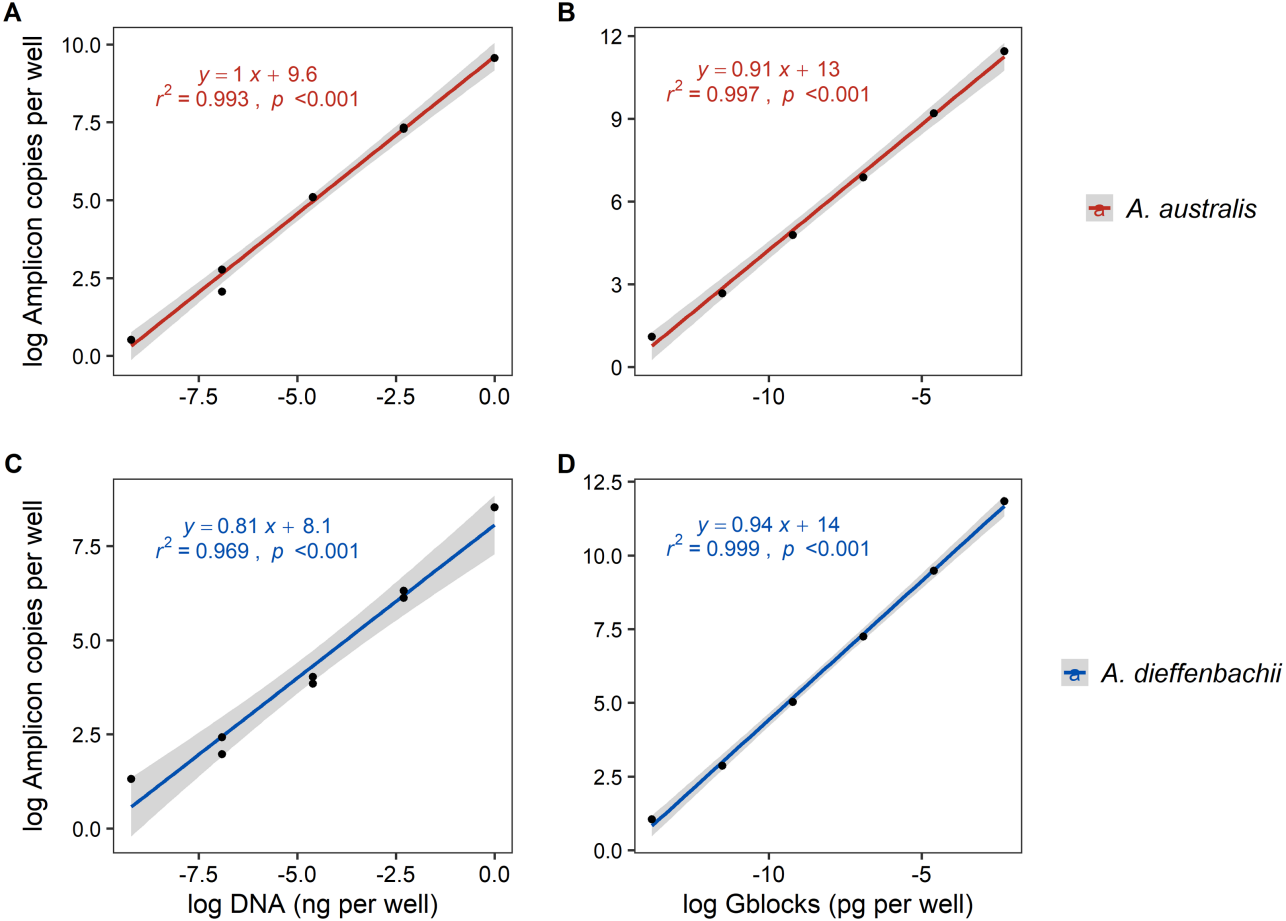
Linear regression analysis of droplet digital PCR copy numbers and target DNA concentrations from eel tissue DNA or synthetic amplicon sequences. The relationship between the log-transformed amplicon copies per well of droplet digital PCR (A, B) *Anguilla australis* and (C, D) *Anguilla dieffenbachii* assays and log-transformed dilution series concentration of target DNA concentrations sourced from (A, C) eel tissue DNA, and (B, D) synthetic amplicon sequence (gblocks). Results from linear regression analysis are shown.

Serial dilutions of tissue DNA and synthetic DNA (gblocks) had a strong and significant correlation (*r*^2^ > 0.96, *p* < 0.001) to the amplicon copies per well determined by ddPCR fluorescence (Fig. 2). Using synthetic gblocks as template, the assays were linear with positive detections in range from 6000–0.06 copies uL^−1^ and 10,000–0.1 copies uL^−1^ for *A. australis* and *A. dieffenbachii*, respectively. Within this linear range, percentage yield of gblock DNA (number of copies measured/number of copies expected) was on average 80.07% ± 15.36% for the *A. australis* assay and 62.65% ± 7.39% for *A. dieffenbachii* assay.

Using tissue DNA as template, the assays were linear with positive detections in range from 1–0.001 ng µL^−1^ for *A. australis* and *A. dieffenbachii* (Fig. 2.). The LOQ and LOD of tissue DNA amplification was 0.001 and 0.0001 ng µL^−^^1^, respectively, for both *A. australis* and *A. dieffenbachii* assays.

### Environmental sample assessment

#### Water samples

Of the 27 filtered river water samples tested, 16 were positive for *A. australis* and 18 for *A. dieffenbachii* in the metabarcoding and ddPCR analysis (Table 1). In addition, the ddPCR assays detected *A. australis* and *A. dieffenbachii* DNA in three and six samples, respectively in which there was no positive detection in the metabarcoding analysis. At sites with biomass assessment (*n* = 5), sites with eel biomass (W11–W13) also had positive detection of eel DNA by metabarcoding or ddPCR for both species. In addition, for W9 and W10, ddPCR and metabarcoding detected *A. dieffenbachii*, despite no eel biomass recorded. Sequencing confirmed the correct ddPCR amplification of both *A. australis* and *A. dieffenbachii* DNA in sample W5 as well as additional confirmation of *A. australis* DNA in sample W6 (Table 1).

**Figure 3:**
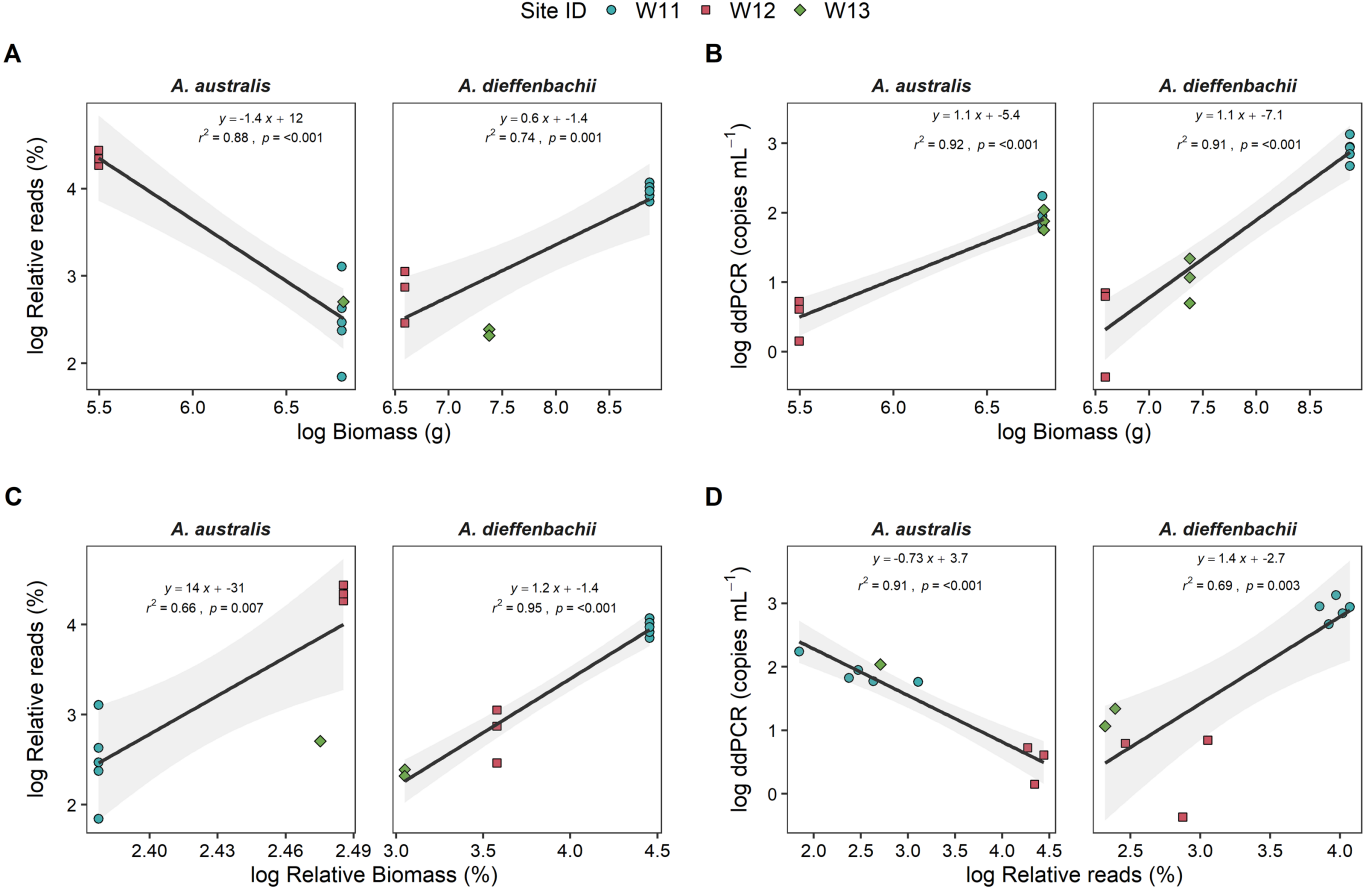
Targeted species-specific linear regressions between droplet digital PCR and metabarcoding analyses of eel DNA in river water and associated eel biomass. The relationships between log-transformed (A) high-throughput sequencing relative reads (%) and eel biomass (g), (B) droplet digital PCR (ddPCR) amplicon copies per mL of filtered water and eel biomass (g) (C) high-throughput relative reads (%) and eel biomass relative to total fish biomass (%) and (D) dd PCR amplicon copies per mL of filtered water and high-throughput sequencing relative reads for *Anguilla dieffenbachii* and *Anguilla australis* at three river sites (*n* = 3 DNA samples per site except for W11 with *n* = 5). Results from linear regression analysis are shown.

Eel biomass at sites differed between species, ranging from 0–901 g and 0–7130 g for *A. australis* and *A. dieffenbachii*, respectively. There was a significant positive relationship (*p* < 0.001) between eel biomass (g) in the river and ddPCR copy numbers per mL of river water filtered for both *A. australis* and *A. dieffenbachii* (Fig. 3). Goodness of fit of models were strong, with high *r*^2^ values for the *A. australis* (*r*^2^ = 0.92) and *A. dieffenbachia* (*r*^2^ = 0.91). For *A. australis*, a positive relationship (*p* < 0.007) but with a lower goodness of fit (*r*^2^ = 0.66) was also identified between metabarcoding relative eel reads (%) and relative biomass, however a contrasting negative relationship was identified when % reads was compared to absolute *A. australis* biomass (g; Fig. 3). In contrast, a significant positive relationship was identified between metabarcoding relative eel reads (%) and both total and relative biomass for *A. dieffenbachia*, with *r*^2^ values improved when relative eel reads were compared to relative biomass (*r*^2^ = 0.74) in comparison to absolute biomass (*r*^2^ = 0.95; Fig. 3). Despite significant relationships existing between eel DNA proxies (ddPCR and metabarcoding relative reads) and eel biomass, there was only a positive relationship between ddPCR copies and relative metabarcoding reads for *A. dieffenbachii* (*r*^2^ = 0.69, *p* = 0.003) with an opposing negative relationship for *A. australis* (*r*^2^ = 0.91, *p* < 0.001; Fig. 3).

There was a proportion of eel biomass and metabarcoding reads that were identified as *Anguilla* sp. but were unable to be further classified to species level. In the metabarcoding analysis of sample W11, *Anguilla* sequences that could only be assigned to genus level accounted for a relatively small proportion of total fish community (1.64 ± 0.03%) in comparison to the proportion of *A. australis* and *A. dieffenbachii* (13% and 53%, respectively). In comparison, *Anguilla* biomass that could not be morphologically identified to species level was 0.8%, 49.2% and 26% of the total eel biomass at sites W11, W12 and W13, respectively. Analysis of species-specific relationships between DNA proxies and biomass did not include unidentified *Anguilla* biomass.

**Figure 4:**
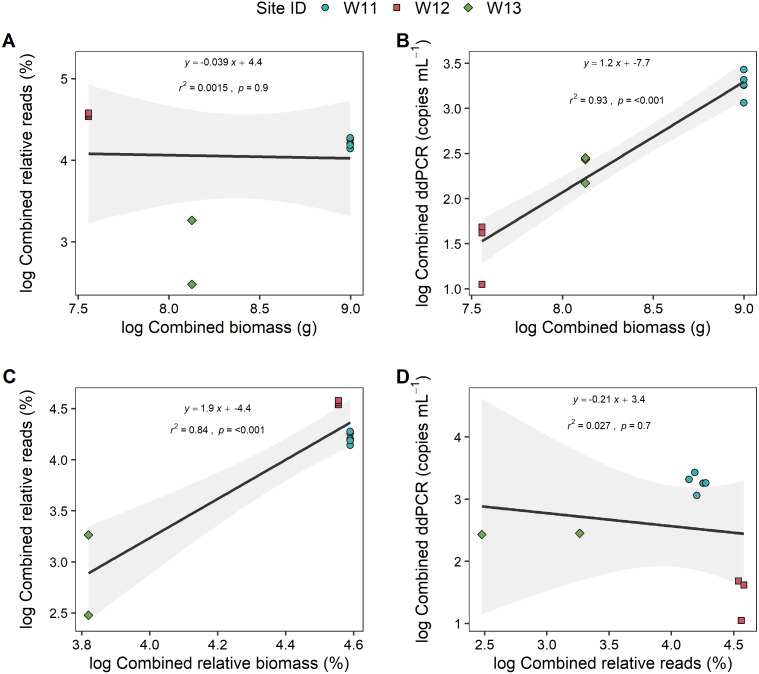
Linear regressions between droplet digital PCR and metabarcoding analyses of total eel DNA in river water and associated eel biomass. The relationships between log-transformed (A) high-throughput sequencing relative reads (%) and eel biomass (g), (B) droplet digital PCR (ddPCR) amplicon copies per mL of filtered water and eel biomass (g) (C) high-throughput relative reads (%) and eel biomass relative to total fish biomass (%) and (D) dd PCR amplicon copies per mL of filtered water and high-throughput sequencing relative reads for *Anguilla* (combined results from *Anguilla dieffenbachii*, *Anguilla australis* and unidentified *Anguilla*) at three river sites (*n* = 3 DNA samples per site except for W11 with *n* = 5). Results from linear regression analysis are shown.

To further explore if the high proportion of unspecified *Anguilla* biomass impacted these relationships, analyses were also carried out at genus level (*i.e.,* combined species data for metabarcoding and ddPCR analyses) that also included unidentified *Anguilla* biomass in the models (Fig. 4). These produced positive, significant (*p* < 0.001) and strong (*r*^2^ > 0.8) relationships between ddPCR concentrations and total biomass as well as between metabarcoding relative reads and relative biomass. No significant relationship was identified between % reads and absolute biomass (*p* = 0.9; Fig. 4). Even with species data combined, there was no significant relationship between ddPCR concentrations and relative metabarcoding relative reads (*r*^2^ = 0.03, *p* = 0.07; Fig. 4).

#### Sediment and biofilm samples

The ddPCR assay results were positive for *A. dieffenbachii* in all samples from Lake Rotoiti, with only one detection of *A. australis* (Table 2). This was consistent with multiple *A. dieffenbachii* observed at the site during sampling. In the Maitai River, *A. dieffenbachii* was detected in only two of the five samples (M1 and M5) even though multiple *A. dieffenbachii* were visibly present at the sampling site. No *A. australis* was detected. There were three and two positive detections for *A. australis* and *A. dieffenbachii* in the Tasman Valley Stream, respectively (Table 2), consistent with the visual identification of both species. Despite both eel species being present, eels were not detected in three out of the five samples from Tasman Valley Stream.

**Table 2:** Droplet digital PCR analysis of *Anguilla* DNA extracted from surface sediments. Droplet digital PCR (ddPCR) amplification with *Anguilla dieffenbachii* and *Anguilla australis* specific assays of DNA extracted from various surface sediment samples.

Site	Sample ID	Species identified	ddPCR (number of copies per mg wet weight of material)	Eels present at sampling (++) abundant (>10) (+) present (>0 and <10) (-) absent
Lake Rotoiti	R1	*A. australis*	0.29^b^	–
		*A. dieffenbachii*	2.81	++
	R2	*A. australis*	–	–
		*A. dieffenbachii*	0.56	++
	R3	*A. australis*	–	–
		*A. dieffenbachii*	3.59	++
	R4	*A. australis*	–	–
		*A. dieffenbachii*	1.32	++
	R5	*A. australis*	–	–
		*A. dieffenbachii* ^a^	3.21	++
Maitai River	M1	*A. australis*	–	–
		*A. dieffenbachii*	0.16^b^	++
	M2	*A. australis*	–	–
		*A. dieffenbachii*	–	++
	M3	*A. australis*	–	–
		*A. dieffenbachii*	–	++
	M4	*A. australis*	–	–
		*A. dieffenbachii*	–	++
	M5	*A. australis*	–	–
		*A. dieffenbachii*	0.25^b^	++
Tasman Valley Stream	J1	*A. australis*	–	+
		*A. dieffenbachii*	0.13^b^	++
	J2	*A. australis*	–	+
		*A. dieffenbachii*	–	++
	J3	*A. australis*	–	+
		*A. dieffenbachii*	–	++
	J4	*A. australis*	–	+
		*A. dieffenbachii*	–	++
	J5	*A. australis* ^a^	40.54	+
		*A. dieffenbachii* ^a^	24.76	++
	J6	*A. australis*	1.07^b^	+
		*A. dieffenbachii*	6.88	++

**Notes.**

a ddPCR products were sequenced and confirmed amplification of correct sequence.

b single droplet samples were measured in triplicate to confirm true positives.

## Discussion

### Assay design, specificity, and sensitivity

In this study we successfully developed ddPCR assays for two closely related eel species, *A. australis* and *A. dieffenbachii*. Using these assays, eDNA from both species was detected in environmental water and sediment samples collected from lakes and rivers. There was no cross-reactivity with any of the other New Zealand freshwater fish species tested. These results corroborate many studies that highlighted the ability of probe-based qPCR or ddPCR assays to specifically detect freshwater fishes including other *Anguilla* sp. in environmental samples, even at low abundances (*e.g.*, Atkinson et al., 2018; Bergman et al., 2016; Itakura et al., 2020; Itakura et al., 2019; Piggott, 2017; Simmons et al., 2015; Weldon et al., 2020).

Attaining species-specific detection can be problematic when attempting to distinguish among closely related species. For example, Wilcox et al. (2013) and Wilcox et al. (2015) noted it was challenging to design species-specific assays for closely related species of char (*Salvelinus* sp.) and subspecies of trout (*Oncorhynchus* sp.), respectively. A decline in assay specificity can result in an increase of both false negative and positive target species detections (Freeland, 2017; Wilcox et al., 2013). In the present study, there was high sequence similarity between *A. dieffenbachii* and *A. australis* for both target genes and therefore careful primer and probe design was required to maximize sequence mismatches between the species. Wilcox et al. (2013) highlighted the importance of mismatches being in the primer in preference to the probe, and for these mismatches to be concentrated at the 3′end of the primers. We followed this approach, which restricted the flexibility of primer and probe design. Although this enabled specific assays to be developed, it is likely that assay sensitivity was slightly reduced (*i.e.,* for optimised ddPCR assay design refer to Edwards & Logan, 2004; Huggett et al., 2013). The LOQ for target tissue DNA was 10^−3^ ng µL^−1^ and LOD 10^−4^ ng µL^−1^, respectively for *A. australis* and *A. dieffenbachii*. These levels are within the ranges of LOD and LOQ reported for other targeted species assays, albeit at the lower end of sensitivity. For example, LOD for the mussel *Margaritifera margaritifera* was 10^−4^ ng or 10^−5^ ng of DNA depending on the target gene (Mauvisseau et al., 2019a; Stoeckle, Kuehn & Geist, 2016), whereas higher sensitivity was found for the invasive crayfish *Procambarus clarkia*, and the endangered newt *Triturus cristatus* (10–8 ng µL^−1^ and 10–7 ng µL^−1^, respectively; Buxton et al., 2017; Tréguier et al., 2014). Despite lower LODs, the LOQs for *A. australis* and *A. dieffenbachii* were similar to *P. clarkia* and *T. cristatus* (10–4 ng µL^−1^ and 10–5 ng µL^−1^, respectively; Buxton et al., 2017; Tréguier et al., 2014).

### Comparison of droplet digital PCR with metabarcoding

Positive eel DNA detection by ddPCR occurred at all sites with eel presence as determined by metabarcoding analysis. Furthermore, the new targeted ddPCR approach resulted in a slightly higher number of positives detections of *A. dieffenbachii* and *A. australis* in comparison to commonly used metabarcoding methods (MiFish-U/E and Teleo-F/R; Miya et al., 2015; Valentini et al., 2016) corroborating the results from other studies (Bylemans et al., 2019; Harper et al., 2018; Schenekar et al., 2020). Primer bias is a plausible explanation for the lower number of detections in the metabarcoding approach. Metabarcoding studies on ‘mock communities’ have highlighted that the detection of specific taxa within more complex communities can be markedly reduced and alluded to primer bias as a reason for this (Lee et al., 2012; Pochon et al., 2013). In a complex freshwater community matrix, as investigated here, the target gene copy numbers of other taxa in the samples may be differentially enhanced in comparison to *A. dieffenbachii* and *A. australis.* These results highlight the need for careful consideration when using metabarcoding approaches to detect specific species in environmental samples.

### Comparison of DNA methods with biomass measurement

Positive DNA detections in the water aligned with the presence of eel biomass at sites. In addition, metabarcoding and ddPCR positively detected eel DNA in the water at a site with no eel biomass measured. This positive detection could be due to various factors, *i.e.,* DNA methods being more sensitive than electrofishing methods that are known to range in efficacy (Meador, McIntyre & Pollock, 2003) or downstream transportation of fish eDNA from above the defined fishing site (Pont et al., 2018).

Both ddPCR and metabarcoding DNA detection methods performed well at estimating *A. dieffenbachii* biomass across five river sites as determined by traditional electrofishing approaches. The ddPCR approach improved model goodness of fit and had a positive significant relationship with *A. australis* biomass in comparison to metabarcoding, suggesting that the relationship with ddPCR concentration was more reliable at a lower biomass, as found for *A. australis*. In previous studies, eDNA concentrations in water samples have been similarly correlated to eel abundance and/or biomass in rivers (Chin et al., 2021; Itakura et al., 2020; Itakura et al., 2019) and lakes (Weldon et al., 2020). Despite this, the reliability of using eDNA concentrations to quantify population abundances is under considerable debate. Some studies on a wider range of organisms have found a positive correlation among results generated using molecular techniques and biomass and abundance estimates (Klobucar, Rodgers & Budy, 2017; Klymus et al., 2015; Mizumoto et al., 2018; Takahara et al., 2012), while others note the absence of such correlation (Capo et al., 2019; Deutschmann et al., 2019; Spear et al., 2015). Many of these positive relationships have been found in controlled laboratory set ups (Doi et al., 2015b; Harper et al., 2019; Klobucar, Rodgers & Budy, 2017; Mizumoto et al., 2018; Takahara et al., 2012) with limited success in the natural environment (Capo et al., 2019; Yates, Fraser & Derry, 2019). There are a number of factors such as temperature (Lacoursière-Roussel, Rosabal & Bernatchez, 2016; Takahara et al., 2012), and feeding and diet (Klymus et al., 2015) that influence the amount of DNA in the environment and thus the relationship between abundance or biomass and eDNA concentrations. Further caution is required as different life stages often have variable cell numbers and different amounts of DNA may be shed at each life stage. For example, Takeuchi et al. (2019) found that concentrations of eDNA shed from the Japanese eel differed significantly among all life stages. In fresh water, the eel life cycle encompasses elvers (ca. 6–20 cm), juveniles and adults (up to 24 kg and 3 kg for *A. dieffenbachii* and *A. australis*, respectively) with sexual dimorphism in body size (Todd, 1980). At each eel life stage there are also differences in habitat as well as diet (*e.g.*, Jellyman, 1996; Jellyman & Chisnall, 1999). Controlled experiments to compare the detection of eel DNA in water and sediment with known parameters such as eel abundance, sex and body size are required to address these issues and understand the future potential of using eel DNA as a proxy for abundance under different conditions. Despite these uncertainties, targeted approaches, such as the ddPCR assays developed in this study are extremely sensitive and specific. The results are obtained instantaneously after the PCR step and using the BioRad machine up to 96 samples including controls can be analyzed simultaneously allowing for high-throughput and rapid turnaround times.

### Application of droplet digital PCRs on surface sediment DNA

Positive eel DNA detection in sediment samples aligned with the presence of eels at sites as determined by visual surveys. However, in contrast to the consistency of water eDNA detections, our data indicates that eDNA detections are more variable in sediment. Several sediment samples were collected at three sites (two rivers, one lake). At each site these were spatially close and taken near target species (ca. 5 m distance). Positive detections (per site) corresponded to the eel species observed at sites, but detections were variable among samples with some replicates failing to detect either eel species, highlighting the problem of false negatives. There is mixed evidence in the literature about the effectiveness of assessing eDNA in sediment. Turner et al. (2014) found that fish DNA persisted for longer in sediment than water and suggested that eDNA was more stable in sediment. In contrast, comparisons between water and sediment samples for targeted fish detection or metabarcoding found that detection was more effective in water column samples (Buxton, Groombridge & Griffiths, 2018; Eichmiller, Bajer & Sorensen, 2014; Shaw et al., 2016). Eichmiller, Bajer & Sorensen (2014) observed that DNA was concentrated in sediment but was highly variable and suggested this was due to differential deposition and resuspension of sediment and DNA degradation. A larger number of samples from a wider variety of habitats are required to confirm these possible explanations. Furthermore, different sampling strategies and sample replication need to be investigated to determine how sampling methods may affect the occurrence of false negatives and therefore the likelihood of positive detection. This next step is necessary before considering the application of these eDNA assays as monitoring tools.

## Conclusions

In this study we successfully developed species-specific ddPCR assays to detect *A. dieffenbachii* and *A. australis* DNA in both water and sediment samples. The ddPCR assays detected eels in a greater number of waters samples than when metabarcoding techniques were applied. Water sample analyses using ddPCR and metabarcoding methods were positively correlated with species-specific biomass. We recommend further research across a greater number and type of river sites to determine the consistency of these relationships and establish whether DNA methods are a reliable proxy of eel biomass. When analyzing surface sediment/biofilm samples, there were several false negative results that may relate to our ability to effectively extract DNA from sediment/biofilms or spatial variation in organism DNA. The successful detections of eel DNA in water by ddPCR in addition to its correlation with eel biomass coupled with the high-throughput and rapid turnaround times highlights the potential for using these assays as a monitoring tools which would enable analysis of eel population at scales and resolutions not previously possible.

##  Supplemental Information

10.7717/peerj.12157/supp-1 Supplemental Information 1 NCBI accession numbers of *Anguilla* gene sequences used to design digital droplet PCR (ddPCR) primer and probe assays for cytochrome b (*cytb*) mitochondrial gene specific to *Anguilla* The target amplicon within *cytb* was a 138 bp region that was 100% similar to *A. dieffenbachii* but maximised interspecific variability among other *Anguilla* species. Accession numbers of other eel species included in alignments to test for *in silico* cross-reactivity are also shown.

10.7717/peerj.12157/supp-2 Supplemental Information 2 NCBI accession numbers of *Anguilla* gene sequences used to design digital droplet PCR (ddPCR) primer and probe assays for 16S ribosocombination of surfmal RNA (16S rRNA) mitochondrial gene specific to *Anguilla australis* The target amplicon within 16S rRNA was a 126 bp region that was 100% similar to *A. australis* but maximised interspecific variability among other *Anguilla* species. Accession numbers of other eel species included in alignments to test for *in silico* cross-reactivity are also shown.

10.7717/peerj.12157/supp-3 Supplemental Information 3 Water samples collected from New Zealand rivers or streams for environmental DNA extraction Water samples (volumes ranged from 0.25–10 L) collected mid-river using a Smith-Root eDNA backpack sampler (eDNA BPS) or Geotech pump system from a range of New Zealand rivers and filtered for environmental DNA extraction. *NA*, not available; *NR*, not relevant.

10.7717/peerj.12157/supp-4 Supplemental Information 4 Surface sediment samples collected from New Zealand freshwater bodies for environmental DNA extraction

10.7717/peerj.12157/supp-5 Supplemental Information 5 Sequence information for synthetic sections of DNA (gblocks) Sequence information for synthetic sections of target DNA (gblocks) designed to include the *A. australis* 16S rRNA gene sequence and the *A. dieffenbachii* cytb gene sequence amplified during ddPCR. Primer and probe sequences for each gene are shown in bold.

10.7717/peerj.12157/supp-6 Supplemental Information 6 GenBank accession numbers for the 12S rRNA gene of New Zealand native freshwater fish species Adapted from Banks, Kelly & Clapcott (2020) showing native New Zealand freshwater fish species sequenced for the 12S rRNA gene and their GenBank accession numbers.

10.7717/peerj.12157/supp-7 Supplemental Information 7 Droplet analysis of *Anguilla australis* and *Anguilla dieffenbachii* droplet digital PCR assays using eel tissue DNA Droplet analysis of; (A) *Anguilla australis* (short-finned eel; SF), and (B) *Anguilla dieffenbachii* (longfinned eel; LF) droplet digital PCR assays (ddPCR). SF DNA, short-finned eel tissue DNA; LF DNA, long-finned eel tissue DNA. NTC, non-template control. Initial eel DNA concentrations in each assay are shown. Positive droplet threshold (pink line) is 2,000 for short-finned eel assay and 10,000 for long-finned eel assay.

## Additional Information and Declarations

### Field Study Permissions

The following information was supplied relating to field study approvals (i.e., approving body and any reference numbers): All tissue samples were collected under the specifications of Special Permit 651 from the New Zealand government agency Ministry for Primary Industries.

### Data Availability

The following information was supplied regarding data availability: The raw sequence reads are available in the NCBI Short Read Archive: PRJNA729802, SRP319777, SRX10876622 SRX10876641.
