# Supplementary material for: Development of droplet digital Polymerase Chain Reaction assays for the detection of long-finned (Anguilla dieffenbachii) and short-finned (Anguilla australis) eels in environmental samples
Source: PeerJ. 2021 Sep 27;9:e12157. doi: 10.7717/peerj.12157 (PMC8483004; doi:10.7717/peerj.12157)
Supplement: Supplemental Information 1 — The target amplicon within cytb was a 138 bp region that was 100% similar to A. dieffenbachii but maximised interspecific variability among other Anguilla species. Accession numbers of other eel species included in alignments to test for in silico cross-reactivity are also shown. [file peerj-09-12157-s001.docx]

**Supplemental Table S1. NCBI accession numbers of *Anguilla* gene sequences used to design digital droplet PCR assays for *Anguilla dieffenbachii.***

National Centre for Biotechnology Information (NCBI) accession numbers of *Anguilla* gene sequences used to design digital droplet PCR (ddPCR) primer and probe assays for cytochrome b (*cytb*) mitochondrial gene specific to *Anguilla dieffenbachii* (New Zealand long-finned eel). The target amplicon within *cytb* was a 138 bp region that was 100% similar to *A. dieffenbachii* but maximised interspecific variability among other *Anguilla* species. Accession numbers of other eel species included in alignments to test for *in silico* cross-reactivity are also shown.

| Species | Common name | NCBI accession numbers | % sequence similarity to target amplicon |
| --- | --- | --- | --- |
| *Anguilla dieffenbachii* | New Zealand long-finned eel | AF006711, AP007240JQ312094-96, AB021770 | 100% |
| *Anguilla australis* | New Zealand short-finned eel | AP007234, AF006712-13, D84303, JQ312086-87, FJ710911-12, AB279340-55, AB021775 | 89% |
| *Anguilla reinhardtii* | Australian long-finned eel | D84301, AB021768, JQ312106-8, AF006706, AP007248 | 90% |
| *Anguilla bicolor pacifica* | Indian short-finned eel | AB021774 | 92% |
| *Anguilla Japonica* | Japanese eel | AB021772 | 92% |
| *Anguilla celebesensis* | Celebes long-finned eel | AB021777 | 92% |
| *Anguilla anguilla* | European eel | AF006714 | 91% |
| *Anguilla rostrata* | American eel | HG794877 | 90% |
| *Anguilla bicolor bicolor* | Indonesian short-finned eel | AF006710 | 92% |
| *Anguilla megastoma* | Polynesian long-finned eel | AB021771 | 92% |
| *Anguilla interioris* | Highlands long-finned eel | AB021773 | 90% |
| *Anguilla obscura* | Pacific short-finned eel | AB021781 | 90% |
| *Anguilla marmorata* | Giant mottled eel | AF485278 | 90% |
| *Anguilla bengalensis* | Mottled eel | KT895265 | 90% |
| *Anguilla*  *luzonensis* | Philippine mottled eel | AB758638, AB758631 | 90% |
| *Anguilla*  *malgumora* | Indonesian long-finned eel | AF006719 | 90% |
