# Supplementary material for: Development of droplet digital Polymerase Chain Reaction assays for the detection of long-finned (Anguilla dieffenbachii) and short-finned (Anguilla australis) eels in environmental samples
Source: PeerJ. 2021 Sep 27;9:e12157. doi: 10.7717/peerj.12157 (PMC8483004; doi:10.7717/peerj.12157)
Supplement: Supplemental Information 2 — The target amplicon within 16S rRNA was a 126 bp region that was 100% similar to A. australis but maximised interspecific variability among other Anguilla species. Accession numbers of other eel species included in alignments to test for in silico cross-reactivity are also shown. [file peerj-09-12157-s002.docx]

**Supplemental Table S2. NCBI accession numbers of *Anguilla* gene sequences used to design digital droplet PCR assays for *Anguilla australis.***

National Centre for Biotechnology Information (NCBI) accession numbers of *Anguilla* gene sequences used to design digital droplet PCR (ddPCR) primer and probe assays for 16S ribosomal RNA (16S rRNA) mitochondrial gene specific to *Anguilla australis* (New Zealand short-finned eel). The target amplicon within 16S rRNA was a 126 bp region that was 100% similar to *A. australis* but maximised interspecific variability among other *Anguilla* species. Accession numbers of other eel species included in alignments to test for *in silico* cross-reactivity are also shown.

| Species | Common name | NCBI accession numbers | % sequence similarity to target amplicon |
| --- | --- | --- | --- |
| *Anguilla dieffenbachii* | New Zealand long-finned eel | AP007240, AB021754 | 86% |
| *Anguilla australis* | New Zealand short-finned eel | AB278692-99, AB278700-24 | 100% |
| *Anguilla reinhardtii* | Australian long-finned eel | DQ645686, AB021761, AJ244824.2, AJ244830 | 91% |
| *Anguilla bicolor pacifica* | Indian short-finned eel | AB021757 | 90% |
| *Anguilla Japonica* | Japanese eel | AB038556, AB021748 | 94% |
| *Anguilla celebesensis* | Celebes long-finned eel | AB021753 | 91% |
| *Anguilla anguilla* | European eel | DQ916152, AB021749 | 90% |
| *Anguilla rostrata* | American eel | AB021759 | 89% |
| *Anguilla bicolor bicolor* | Indonesian short-finned eel | AP007236 | 90% |
| *Anguilla megastoma* | Polynesian long-finned eel | AB021758 | 94% |
| *Anguilla interioris* | Highlands long-finned eel | AB021764 | 91% |
| *Anguilla obscura* | Pacific short-finned eel | AP007247 | 90% |
| *Anguilla marmorata* | Giant mottled eel | DQ093412 | 90% |
| *Anguilla bengalensis* | Mottled eel | AP007245 | 90% |
| *Anguilla*  *luzonensis* | Philippine mottled eel | HQ197930, AB469437 | 90% |
| *Anguilla*  *malgumora* | Indonesian long-finned eel | AB021752 | 90% |
