# Supplementary material for: Development of droplet digital Polymerase Chain Reaction assays for the detection of long-finned (Anguilla dieffenbachii) and short-finned (Anguilla australis) eels in environmental samples
Source: PeerJ. 2021 Sep 27;9:e12157. doi: 10.7717/peerj.12157 (PMC8483004; doi:10.7717/peerj.12157)
Supplement: Supplemental Information 3 — Water samples (volumes ranged from 0.25–10 L) collected mid-river using a Smith-Root eDNA backpack sampler (eDNA BPS) or Geotech pump system from a range of New Zealand rivers and filtered for environmental DNA extraction. NA, not available; NR, not relevant. [file peerj-09-12157-s003.docx]

| Site ID | Sampling Date | Sampling Site | | | | Sampling method | Filter size (µm) | Filtered volume (L) | Sample size | Biomass data |
| --- | --- | --- | --- | --- | --- | --- | --- | --- | --- | --- |
|  |  | Region | Site Name | Latitude | Longitude |  |  |  |  |  |
| W1 | March 2019 | Tasman | Orphanage Stream | 41°19'36.5"S | 173°12'59.0"E | Geotech | 1.2 | *NA* | *n = 1* | *No* |
| W2 | April 2019 | Waikato | Komakorau Stream | 37°38'56.3"S | 175°15'34.7"E | Geotech | 1.2 | 0.4 | *n = 1* | *No* |
| W3 | May 2019 | Tasman | Riwaka River tributary | 41°04'32.0"S | 172°59'42.6"E | eDNA BPS | 1.2 | 1 | *n = 1* | *No* |
| W4 | May 2019 | Tasman | Motueka River tributary | 41°08'17.4"S | 172°58'42.6"E | eDNA BPS | 1.2 | 0.25 | *n = 1* | *No* |
| W5 | May 2019 | Tasman | Moutere River | 41°09'49.5"S | 172°59'35.6"E | eDNA BPS | 5 | 10 | *n = 1* | *No* |
| W6 | May 2019 | Tasman | Moutere River | 41°08'45.0"S | 173°00'01.2"E | eDNA BPS | 5 | 4.44 | *n = 1* | *No* |
| W7 | June 2019 | Nelson | Brook Stream | 41°18'43.8"S | 173°17'31.5"E | eDNA BPS | 5 | *NA* | *n = 1* | *No* |
| W8 | June 2019 | Nelson | Brook Stream | 41°18'43.8"S | 173°17'31.5"E | eDNA BPS | 1.2 | *NA* | *n = 3* | *No* |
| W9 | January 2020 | Canterbury | Little Joseph Creek | 43°51'29.9"S | 170°26'27.8"E | eDNA BPS | 5 | 3.69, 3.9, 3.15 | *n = 3* | *Yes* |
| W10 | January 2020 | Canterbury | Upper Irishmans Creek | 43°58'37.6"S | 170°19'46.0"E | eDNA BPS | 5 | 4.74, 3.97, 5.14 | *n = 3* | *Yes* |
| W11 | January 2020 | Nelson | Poorman Valley Stream | 41°18'10.4"S | 173°13'32.1"E | eDNA BPS | 5 | 5, 5.01, 5, 5, 5 | *n = 5* | *Yes* |
| W12 | March 2020 | Tasman | Reservoir Creek | 41°20'36.0"S | 173°12'07.0"E | eDNA BPS | 5 | 5.09, 5.39, 5.42 | *n = 3* | *Yes* |
| W13 | March 2020 | Tasman | Lower Moutere River | 41°09'48.2"S | 172°59'35.2"E | eDNA BPS | 5 | 4.36, 4.37, 4.43 | *n = 3* | *Yes* |
| W14 | April 2019 | NA | *NR* (field control) | *NR* | *NR* | eDNA BPS | 1.2 | 1 | *n = 1* | *No* |

**Supplemental Table S3. Water samples collected from New Zealand rivers or streams for environmental DNA extraction**.

Water samples (volumes ranged from 0.25 – 10 L) collected mid-river using a Smith-Root eDNA backpack sampler (eDNA BPS) or Geotech pump system from a range of New Zealand rivers and filtered for environmental DNA extraction. *NA* = not available, *NR* = not relevant.
