# Supplementary material for: Development of droplet digital Polymerase Chain Reaction assays for the detection of long-finned (Anguilla dieffenbachii) and short-finned (Anguilla australis) eels in environmental samples
Source: PeerJ. 2021 Sep 27;9:e12157. doi: 10.7717/peerj.12157 (PMC8483004; doi:10.7717/peerj.12157)
Supplement: Supplemental Information 4 [file peerj-09-12157-s004.docx]

**Supplemental Table S4. Surface sediment samples collected from New Zealand freshwater bodies for environmental DNA extraction.**

| Sampling date | Sampling Site | | | Sample ID | Source Material |
| --- | --- | --- | --- | --- | --- |
|  | Name | Latitude | Longitude |  |  |
| 4 January 2020 | Lake  Rotoiti | 41°48'24.0"S | 172°50'38.1"E | R1 | Sediment biofilm |
|  |  |  |  | R2 | Sediment biofilm |
|  |  |  |  | R3 | Sediment biofilm |
|  |  |  |  | R4 | Sediment/gravel |
|  |  |  |  | R5 | Sediment/gravel |
| 16 January 2020 | Maitai  River | 41°16'47.6"S | 173°19'47.5"E | M1 | Sediment |
|  |  |  |  | M2 | Sediment |
|  |  |  |  | M3 | Sediment |
|  |  |  |  | M4 | Benthic biofilm (algae) |
|  |  |  |  | M5 | Benthic biofilm (algae) |
| 26 January 2020 | Tasman Valley Stream | 41°11'44.6"S | 173°03'25.2"E | J1 | Sediment/gravel |
|  |  |  |  | J2 | Sediment/gravel |
|  |  |  |  | J3 | Sediment/gravel |
|  |  |  |  | J4 | Sediment/gravel |
|  |  |  |  | J5 | Biofilm (off rocks) |
|  |  |  |  | J6 | Biofilm (off rocks) |
