# Supplementary material for: Development of droplet digital Polymerase Chain Reaction assays for the detection of long-finned (Anguilla dieffenbachii) and short-finned (Anguilla australis) eels in environmental samples
Source: PeerJ. 2021 Sep 27;9:e12157. doi: 10.7717/peerj.12157 (PMC8483004; doi:10.7717/peerj.12157)
Supplement: Supplemental Information 5 — Sequence information for synthetic sections of target DNA (gblocks) designed to include the A. australis 16S rRNA gene sequence and the A. dieffenbachii cytb gene sequence amplified during ddPCR. Primer and probe sequences for each gene are shown in bold. [file peerj-09-12157-s005.docx]

**Supplemental Table S5. Sequence information for synthetic sections of DNA (gblocks).**

Sequence information for synthetic sections of target DNA (gblocks) designed to include the *A. australis* 16S rRNA gene sequence and the *A. dieffenbachii* cytb gene sequence amplified during ddPCR. Primer and probe sequences for each gene are shown in bold.

| Species | Gene | gblock DNA sequence (5’–3’) | Sequence length (bp) |
| --- | --- | --- | --- |
| *Anguilla australis* | 16S rRNA | GGACAAAATTATCTCAGTGGG**CCCAAAAGCAGCCACCTG**T**AAAGAAAGCGTTAAAGCTCCGA**TAATAACATTGCAAAAAATAAGA**TAATAAACTCCCCACCCCCT**AAAAATATTAAGCTATCCTAT | 126 |
| *Anguilla dieffenbachii* | *cytb* | TAACCC**GATTCTTCGCATTCCACTTCTTA**TTCCCTATTGTAGTACGTGGAGCTACAATAATAGATCTCCTAT**TCCTACATGAAACAGGATCAAGCAATCCA**GTAGGATT**AAACTCTGACGCAGACAAAGTCC**CATTCC | 138 |
