# Supplementary material for: Development of droplet digital Polymerase Chain Reaction assays for the detection of long-finned (Anguilla dieffenbachii) and short-finned (Anguilla australis) eels in environmental samples
Source: PeerJ. 2021 Sep 27;9:e12157. doi: 10.7717/peerj.12157 (PMC8483004; doi:10.7717/peerj.12157)
Supplement: Supplemental Information 6 — Adapted from Banks, Kelly & Clapcott (2020) showing native New Zealand freshwater fish species sequenced for the 12S rRNA gene and their GenBank accession numbers. [file peerj-09-12157-s006.docx]

**Supplemental Table S6. GenBank accession numbers for the 12S rRNA gene of New Zealand native freshwater fish species.**

Adapted from Banks et al. 2020 showing native New Zealand freshwater fish species sequenced for the 12S rRNA gene and their GenBank accession numbers.

| Species | GenBank accession number |
| --- | --- |
| *Galaxias* aff. *paucispondylus* | MT952795 |
| *Galaxias* aff. *paucispondylus* | MT952796 |
| *Galaxias divergens* | MT952797 |
| *Galaxias divergens* | MT952798 |
| *Galaxias eldoni* | MT952799 |
| *Galaxias eldoni* | MT952800 |
| *Galaxias fasciatus* | MT952801 |
| *Galaxias fasciatus* | MT952802 |
| *Galaxias macronasus* | MT952803 |
| *Galaxias* n sp Waitaki Alpine | MT952804 |
| *Galaxias* n sp Waitaki alpine | MT952805 |
| *Galaxias* northern flathead | MT952806 |
| *Galaxias northern flathead* | MT952807 |
| *Galaxias paucispondylus* | MT952808 |
| *Galaxias paucispondylus* | MT952809 |
| *Galaxias paucispondylus* | MT952810 |
| *Galaxias prognathus* | MT952811 |
| *Galaxias prognathus* | MT952812 |
| *Galaxias pullus* | MT952813 |
| *Galaxias pullus* | MT952814 |
| *Galaxias vulgaris* | MT952815 |
| *Galaxias vulgaris* | MT952816 |
| *Gobiomorphus aff. breviceps* | MT952817 |
| *Gobiomorphus aff. breviceps* | MT952818 |
| *Gobiomorphus breviceps* | MT952819 |
| *Neochanna burrowsius* | MT952820 |
| *Neochanna burrowsius* | MT952821 |
| *Neochanna rekohua* | MT952822 |
| *Neochanna rekohua* | MT952823 |
| *Galaxias argenteus* | MT952824 |
| *Galaxias argenteus* | MT952825 |
| *Galaxias postvectis* | MT952826 |
| *Galaxias argenteus* | MT952827 |
| *Gobiomorphus basalis* | MT952828 |
| *Gobiomorphus breviceps* | MT952829 |
| *Gobiomorphus breviceps* | MT952830 |
| *Gobiomorphus gobioides* | MT952831 |
| *Gobiomorphus hubbsi* | MT952832 |
| *Gobiomorphus huttoni* | MT952833 |
| *Gobiomorphus huttoni* | MT952834 |
| *Forsterygion nigripenne* | MT952835 |
| *Forsterygion nigripenne* | MT952836 |
| *Rhombosolea retiaria* | MW187727 |
| *Neochanna apoda* | MW187728 |
| *Scardinius erythrophthalmus* | MW187726 |
| *Cheimarrichthys fosteri* | MW187731 |
| *Cheimarrichthys fosteri* | MW187730 |
