# Supplementary figures and images for: Development of droplet digital Polymerase Chain Reaction assays for the detection of long-finned (Anguilla dieffenbachii) and short-finned (Anguilla australis) eels in environmental samples
Source: PeerJ. 2021 Sep 27;9:e12157. doi: 10.7717/peerj.12157 (PMC8483004; doi:10.7717/peerj.12157)

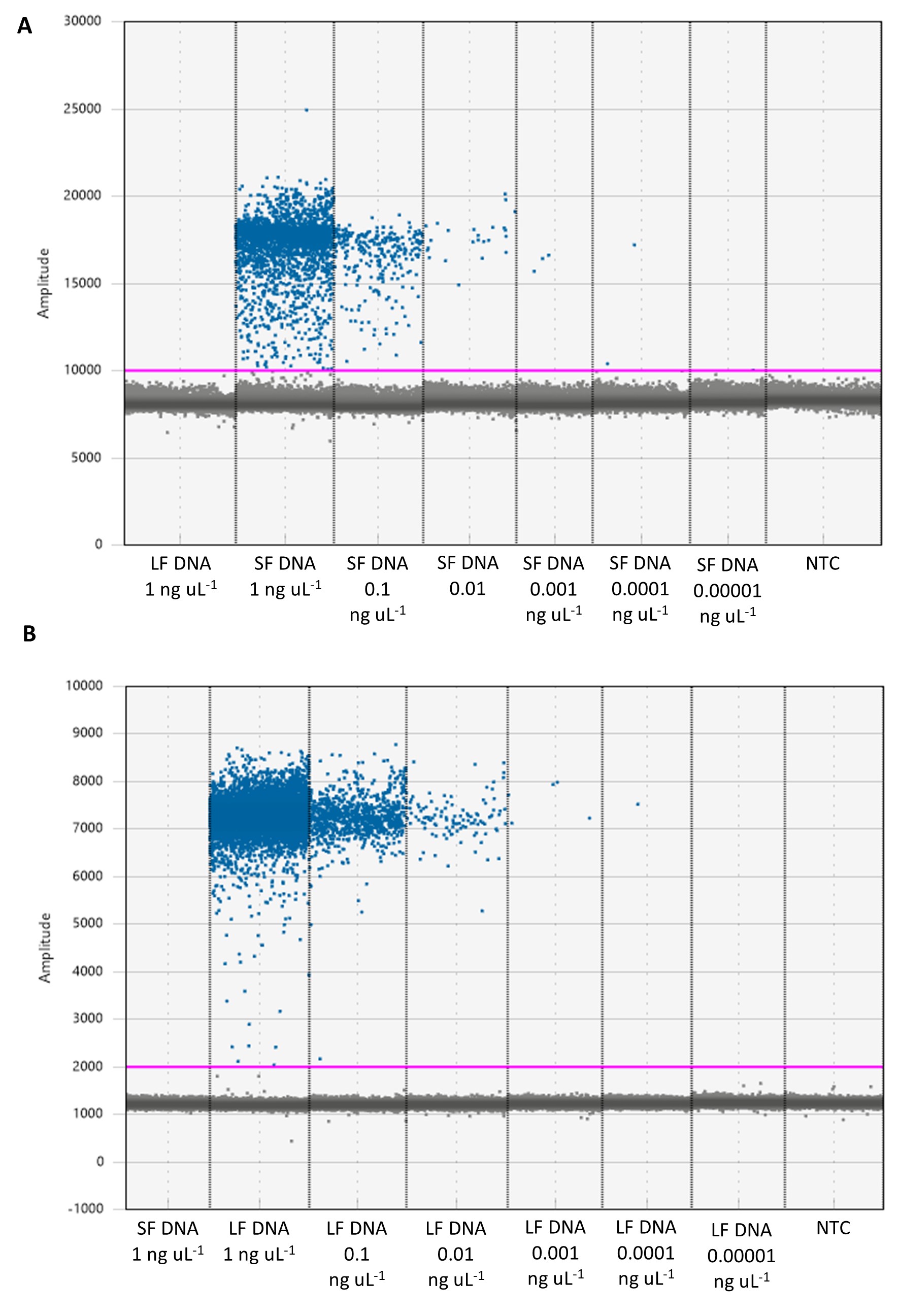

Supplement: Supplemental Information 7 — Droplet analysis of; (A) Anguilla australis (short-finned eel; SF), and (B) Anguilla dieffenbachii (longfinned eel; LF) droplet digital PCR assays (ddPCR). SF DNA, short-finned eel tissue DNA; LF DNA, long-finned eel tissue DNA. NTC, non-template control. Initial eel DNA concentrations in each assay are shown. Positive droplet threshold (pink line) is 2,000 for short-finned eel assay and 10,000 for long-finned eel assay. [file peerj-09-12157-s007.jpg]
